# National physician survey on glycemic goals and medical decision making for patients with type 2 diabetes

**DOI:** 10.1097/MD.0000000000018491

**Published:** 2019-12-20

**Authors:** Neda Laiteerapong, Sandra A. Ham, Aviva G. Nathan, Robert M. Sargis, Michael T. Quinn, Elbert S. Huang

**Affiliations:** a Section of General Internal Medicine, Department of Medicine; b Center for Health and Social Sciences, University of Chicago; c University of Illinois at Chicago, Chicago, IL.

**Keywords:** glycemic control, individualization, medical decision making, physician, type 2 diabetes

## Abstract

To describe how patient characteristics influence physician decision-making about glycemic goals for Type 2 diabetes.

2016 survey of 357 US physicians. The survey included two vignettes, representing a healthy patient and an unhealthy patient, adapted from a past survey of international experts and a factorial design vignette that varied age, heart disease history, and hypoglycemia history. Survey results were weighted to provide national estimates.

Over half (57.6%) of physicians recommended a goal HbA1c <7.0% for most of their patients. For the healthy patient vignette, physicians recommended a goal similar to that of international experts (<6.66% (95% Confidence Interval (CI), 6.61–6.71%) vs <6.5% (Interquartile range (IQR), 6.5–6.8%)). For the unhealthy patient, physicians recommended a lower goal than international experts (<7.38% (CI, 7.30–7.46) vs <8.0% (IQR, 7.5–8.0%)). In the factorial vignette, physicians varied HbA1c goals by 0.35%, 0.06%, and 0.28% based on age, heart disease history, and hypoglycemia risk, respectively. The goal HbA1c range between the 55-year-old with no heart disease or hypoglycemic events and the 75-year-old with heart disease and hypoglycemic events was 0.65%.

Despite guidelines that recommend HbA1c goals ranging from <6.5% to <8.5%, US physicians seem to be anchored on HbA1c goals around <7.0%.

## Abstract 2

Supplemental Digital Content is available in the text

## 1 Introduction

In recent years, the argument for managing patients based on their individualized risk has been gaining momentum. Since 1994, the American Diabetes Association (ADA) has recommended a HbA1c<7.0% goal alongside recommendations to individualize glycemic goals.^[1]^ The impetus to individualize glycemic goals became stronger after the 2008 publication of the ACCORD trial that demonstrated an increased risk of mortality in the study arm randomized to intensive glycemic control.^[2]^ Also, a US-based cost-effectiveness analysis found that when compared to a uniform HbA1c<7.0% goal, individualized HbA1c goals produced significant savings and were associated with increased quality of life.^[3]^ However, to date, the process by which physicians use to select glycemic goals for patients with type 2 diabetes is unclear. Thus, we sought to understand the physician decision-making process when choosing glycemic goals in a national sample of U.S. physicians.

## 2 Methods

### 2.1 Study design

We designed a mail survey about type 2 diabetes care practices for a national sample of primary care and endocrinology physicians (Online-Only Supplement). The majority of questions originated from a survey we had conducted regionally.^[4]^ We developed the survey with input from local experts in health sciences research and diabetes. The survey underwent cognitive testing using the “think aloud” method with practicing physicians and iterative revisions.^[5]^ The survey was mailed in three waves between May and August 2016; $10 incentive was provided with the first wave and a postcard reminder was sent prior to the third wave. This study was approved by the University of Chicago Biological Sciences Division/University of Chicago Medical Center Institutional Review Board.

### 2.2 Survey participants

We mailed the survey to randomly selected physicians who self-identified as practicing in a primary care (N = 720) or endocrinology (N = 480) practice area in the American Medical Association Physician Masterfile. The physician population was stratified into four strata for sampling: by Affordable Care Act expansion state status (yes or no) and specialty (primary care or endocrinology). Stratification by expansion state status was included because of a substudy analyzing associations between expansion state status and diabetes care.^[6]^ We oversampled endocrinologists to increase the response rate for this group. We excluded responses from physicians who reported that they did not provide longitudinal ambulatory care for patients with type 2 diabetes (e.g., hospitalists, nursing home physicians) and physicians who were reported to be deceased or retired (Supplemental Fig. 1).

### 2.3 HbA1c goal-setting

We asked physicians, “In general, what HbA1c goal do you usually recommend for most patients with type 2 diabetes?” (“<6.5%”, “<7.0%”, “<8.0%”, “depends on patient's characteristics”, or “other”). We also asked physicians to select the top 3 patient factors they take into account when managing a patient's HbA1c control. We listed patient characteristics that ADA diabetes care guidelines recommended physicians consider when individualizing HbA1c goals: age, duration of diabetes, life expectancy, history of diabetic complications, history of non-diabetes-related diseases, risk of hypoglycemia from treatment, cognitive function, adherence and motivation, medication costs, and resources and support.^[7]^

We also asked how frequently they individualized HbA1c goals (“never”, “rarely”, “sometimes”, “most of the time”, “always”) and how challenging it was to individualize goals (“not at all”, “slightly”, “moderately”, “very”, “extremely”). If respondents reported that individualization was challenging, we asked physicians to indicate all reasons they found individualizing HbA1c goals to be challenging. We provided nine possible explanations (“Recommendations are conflicting”, “Recommendations are vague”, “I am very often missing duration of diabetes information”, “I lack access to tools to help me estimate life expectancy”, “I lack access to tools to help me estimate hypoglycemia risk”, “There is not enough time to explain individualized goals to patients”, “Patients may perceive their care as suboptimal if their goal is raised”, “Patients are confused by individualized goals”, “Patients prefer a HbA1c level different from their individualized goal”) and a free-response option.

### 2.4 Clinical vignettes

Each survey also included 3 hypothetical clinical vignettes randomly selected out of a pool of 10 vignettes (Table 1).^[8]^ All surveys included one previously published vignette (Table 1, Vignettes 1 and 2) and 2 vignettes from a 2^3^ factorial design (Table 1, Vignettes 3–10).

**Table 1:**
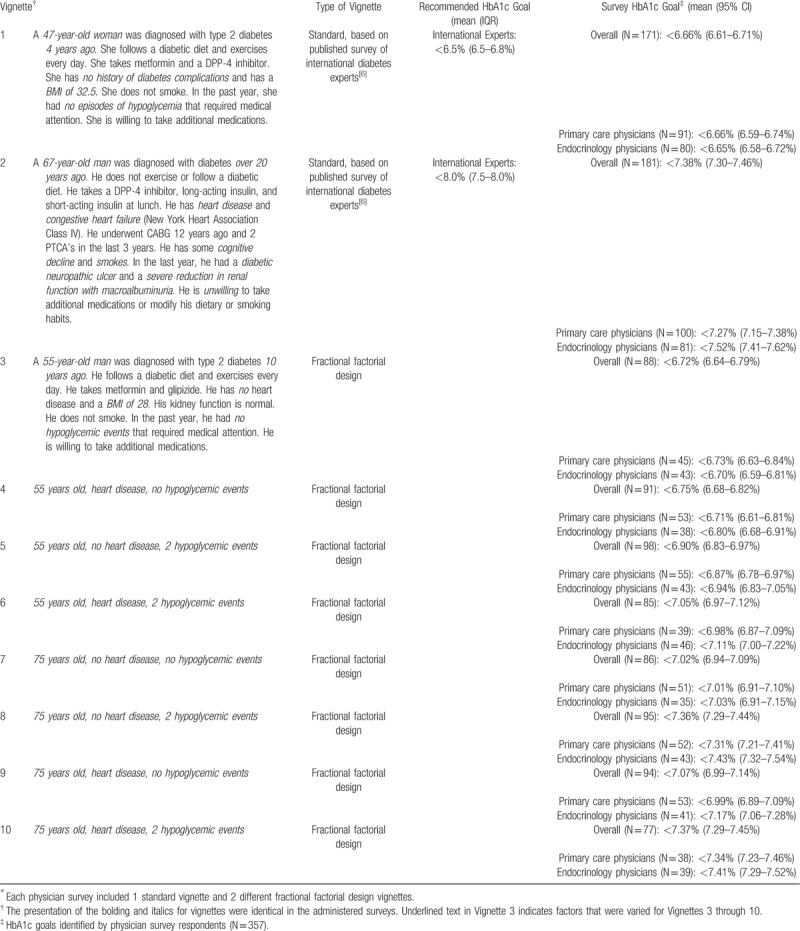
Clinical Vignette and Associated HbA1c Goal^∗^.

The published vignettes originated from a study of international diabetes experts designed to understand the factors that experts used when setting glycemic goals.^[8]^ We selected the 2 published vignettes that the experts assigned the lowest and highest glycemic goals. The lowest glycemic goal was assigned to a patient who had diabetes for 4 years and no complications (Table 1, Vignette 1). This “healthy” patient was assigned a median HbA1c goal of <6.5% (Interquartile range (IQR), 6.5%–6.8%) by experts. The highest glycemic goal was assigned to a patient with several diabetic complications (Vignette 2). This “unhealthy” patient was assigned a median glycemic goal of <8.0% (IQR, 7.5–8.0%) by experts. We modified these vignettes slightly by removing potentially biasing variables (e.g., marital status, occupation, family relationships). We were unable to include the third vignette because of space limitations.

In addition to the standard vignette, each survey included 2 different versions of a vignette with a factorial design (Vignettes 3–10). This design was used to vary three factors across each vignette: age, heart disease history, and hypoglycemia history. We chose these factors because the survey of international experts weighted these factors highly in their determination of goals.^[8]^ We chose numbers for age and hypoglycemia history that may present some clinical uncertainty. Age was either 55- or 75-years old. Heart disease history was either present or absent, and hypoglycemia history was either no events or 2 events requiring medical attention in the last year. Other patient factors (e.g., gender, duration of diabetes, diabetes treatments, body mass index, and willingness to take medications) were the same for all factorial vignettes.

### 2.5 Main outcome

After each vignette, we asked physicians, “What glycemic target would you aim for?” and presented them with a visual analog scale to indicate their response. Our scale had anchors of <6.0%, <7.0%, <8.0%, and <9.0% and vertical marks to indicate 0.25% increments.

### 2.6 Covariates

The survey included questions on physician age, gender, race/ethnicity, specialty, practice setting (hospital-based clinic or other clinic), practice type (single-specialty or multi-specialty), percentage of patients with managed care (0%–50% or 51%–100%), and percentage of patients age 65 years or older (0%–50% or 51%–100%).

#### 2.6.1 Analysis

Survey data for physician characteristics for choice of usual glycemic goals and goals selected for vignettes were summarized using means and percentages. To assess differences in goals and interactions between age, heart disease history, and hypoglycemia history (Vignettes 3–10), unweighted linear mixed regression models were used with a random effect for physician. To account for the survey sample design, post-stratification weights were computed to the 2016 US physician population by the sample design criteria of state Affordable Care Act expansion status (expansion/waiver state, not expansion state) and physician specialty (primary care, endocrinology). All survey results were weighted. Subgroup analyses by physician type (PCP vs endocrinologist) and gender were also conducted. *P* values less than .05 were considered statistically significant. SAS version 9.4 (SAS Institute Inc.) was used to perform analyses.

## 3 Results

### 3.1 Respondent characteristics

Our adjusted response rate was 41% (N = 359) (Supplemental Fig. 1). Two respondents were excluded because they did not answer questions about individualized HbA1c goals. Physician and practice characteristics overall and by usual HbA1C goals are presented in Supplemental Table 1. Fifty-four percent (n = 193) of eligible respondents were primary care physicians; 46% (n = 164) were endocrinologists.

### 3.2 Usual glycemic goal

In the US, over half of physicians (weighted, 57.6%; n = 197) recommended a goal HbA1c <7.0%, about 1 in 5 (weighted, 19.1%; n = 59) recommended a goal HbA1c <6.5%, and 13.1% recommended an individualized goal (n = 79) for most of their patients with type 2 diabetes (Table 2). Only 1 in 10 (weighted, 10.2%; n = 22) of physicians recommended a goal HbA1c <7.5% or <8.0%.

**Table 2:**
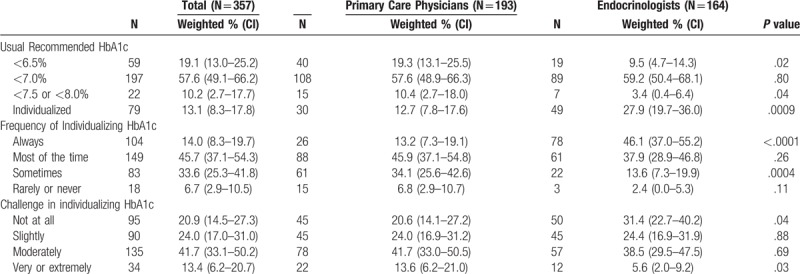
Prevalence estimates of usual recommended HbA1c and factors involved in individualizing, by specialty type, National Physician Survey of Type 2 Diabetes Care Practices, 2016.

Similar percentages of primary care physicians (weighted, 57.6%) and endocrinologists (weighted, 59.2%) recommended an HbA1c goal of <7.0% (*P* = .80). However, endocrinologists were more likely to recommend individualized goals than primary care physicians (weighted, 27.9% vs 12.7%, *P* = .0009), and endocrinologists were less likely to recommend an HbA1c goal of <6.5% (weighted, 9.5% vs 19.3%, *P* = .02).

Endocrinologists were more likely to report “always” (weighted, 46.1% vs 13.2%, *P* < .0001; Table 2) and less likely to report “sometimes” individualizing goals (weighted, 13.6% vs 34.1%, p = 0.0004) than primary care physicians. Just over half of primary care physicians (weighted, 55.3%) and less than half (weighted, 44.1%) of endocrinologists reported that it was “moderately” or “very or extremely” challenging to individualize HbA1c goals. Endocrinologists were less likely than primary care physicians to report individualizing HbA1c goals was “not at all” challenging (weighted, 31.4% vs 20.6%, *P* = .04).

The 3 most common reasons that primary care physicians reported individualizing HbA1c to be challenging were: insufficient time to explain individualized goals (weighted, 31.1%; n = 57), conflicting recommendations (N = 52, 27.3%), and patient confusion regarding individualized goals (N = 51, 26.3%). Endocrinologists also reported that individualizing HbA1c goals was challenging because of patient confusion (weighted, 24.4%; n = 37), a lack of access to tools to estimate hypoglycemia risk (weighted, 24.2%; n = 40), and vague recommendations (weighted, 21.1%; n = 34). Primary care physicians were more likely to report that insufficient time and conflicting recommendations made individualizing goals challenging, while endocrinologists were more likely to report that a patient perception that care is suboptimal if goals are raised, and patient preferences for a level of HbA1c control different than individualized goals, made individualizing goals challenging.

The top 3 patient factors that physicians considered when managing HbA1c were risk of hypoglycemia from treatment (weighted 59.5%; n = 234), history of diabetic complications (weighted, 53.3%; n = 169), and patient age (weighted, 49.3%; n = 166) (Table 3). Primary care physicians more frequently considered history of diabetic complications (weighted, 53.6% vs 40.4%, *P* = .04), medication costs (weighted, 27.3% vs 15.0%, *P* = .02), and resources and support (weighted, 8.7% vs 2.3%, *P* = .01) than endocrinologists, whereas endocrinologists more frequently considered risk of hypoglycemia (weighted, 72.9% vs 59.2%, *P* = .03) and life expectancy (weighted, 51.2% vs 27.6%, *P* < .0001) than primary care physicians.

**Table 3:**
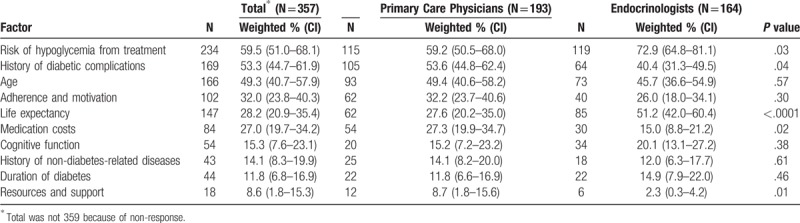
Prevalence estimates of the top factors considered when managing a patient's HbA1c, by physician type, National Physician Survey of Type 2 Diabetes Care Practices, 2016.

### 3.3 Clinical vignettes

For the healthy patient vignette, physicians recommended a mean HbA1c goal similar to experts (Table 1, Vignette 1), (mean, <6.66% (CI, 6.59%–6.73%) vs <6.5% (IQR, 6.5%–6.8%)). However, for the unhealthy patient vignette (Table 1, Vignette 2), physicians recommended a lower goal than experts (<7.34% (CI, 7.31%–7.44%) vs <8.0% (IQR, 7.5%–8.0%)).

**Figure 1:**
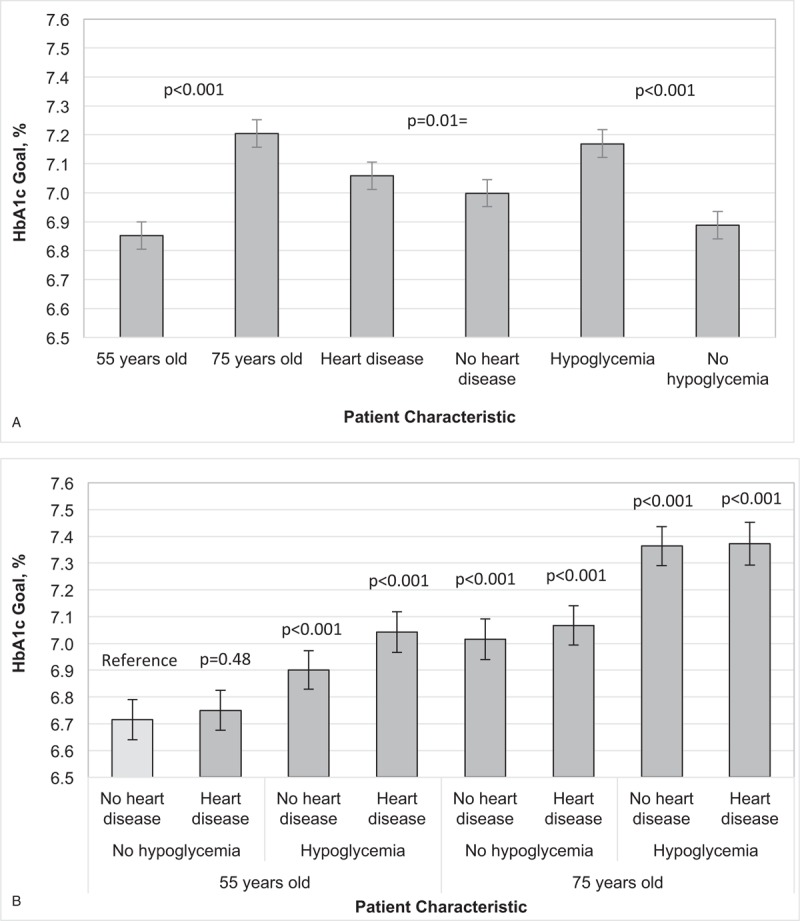
Variation in HbA1c goals by patient age, heart disease history, and hypoglycemia risk, overall and by physician type, national physician survey of Type 2 Diabetes Care Practices, 2016. A. Overall main effects. ^∗∗^p < 0.001. ^∗^*P* < .01. B. Overall interaction effects. ^∗∗^*P* < .0001 vs patient age 55 years old with no heart disease and no severe hypoglycemia in the last year.

For the factorial design vignettes, physicians assigned differed HbA1c goals on the basis of patient age, heart disease history, and hypoglycemia risk (Fig. 1). Since each physician responded to 2 vignettes, each vignette had 77 to 98 responses overall and 35 to 55 responses from physicians of each specialty.

HbA1c goals ranged 0.35% by patient age (55-year-old vs 75-year old: <6.85% (CI, 6.81% to 6.90%) vs <7.20% (CI, <7.16% to <7.25%), *P* < .001) and 0.28% by hypoglycemia history (no recent events vs 2 severe events per year: <6.89% (CI, <6.84% to <6.93%) vs <7.17% (CI, <7.12% to <7.22%), *P* < .001) (Fig. 1). There was a slight difference in HbA1c goals by heart disease history (absent vs present: <7.00% (CI, <6.95% to <7.05%) vs <7.06% (CI, <7.01% to <7.11%), *P* < .01) (Fig. 1A). In analyses that accounted for interactions between age, hypoglycemia risk, and heart disease history, the largest difference in HbA1c goals was 0.65% between the 55-year old with no hypoglycemic episodes or heart disease and the 75-year old with 2 hypoglycemic episodes in the last year and heart disease (Fig. 1B). There were no differences in HbA1c goals by patient age, hypoglycemia risk, or heart disease history by physician specialty (primary care or endocrinology) or gender.

## 4 Discussion

In this national sample of primary care and endocrinology physicians, over half of physicians reported that they chose a goal HbA1c<7.0% for most of their patients. Additionally, in clinical vignettes, this tendency to choose an HbA1c goal of <7.0% was present even for patients who varied greatly in age, heart disease history, and hypoglycemia risk.

Our results suggest that physicians select HbA1c goals close to 7.0%. Our results contrast with a prior survey of physicians at an academic medical center which found that their providers individualized glycemic goals similarly to international experts and their proposed algorithm.^[9]^ A major difference is our study is that we included a national sample of physicians, which likely practice differently than academic physicians. Our results do align with an international randomized trial of individualizing treatment targets among elderly patients with type 2 diabetes that found that the average individualized target was 7.0%.^[10]^ Two potential explanations for the selection of HbA1c goals close to 7.0% include that current guidelines are vague and therefore, physicians reasonably choose a goal around 7%, which was the first HbA1c goal mentioned in the American Diabetes Association care guidelines and has been present in the guidelines since 1994.^[1]^ In addition to the care guideline emphasis on the HbA1c <7.0% goal, this goal has been codified as a HEDIS quality measure by the National Committee on Quality Assurance, which solidified the importance of achieving this goal for physicians and health systems across the U.S. However, the potential harm of HbA1c goals very close to <7.0% is great, especially because achieving this level of HbA1c control often relies on the use of insulin or sulfonylureas, which confers a high risk for hypoglycemia.^[11]^

The vast majority of our physicians reported that they prioritized the same clinical variables (risk for hypoglycemia from treatment, history of diabetic complications, and patient age) that we varied in our clinical vignettes. Interestingly, in our sample of physicians, heart disease history was associated with the least variation in HbA1c (range 0.06% vs 0.28% for severe hypoglycemia history (no vs 2 events in the last year) and 0.35% for age (55 vs 75 years old)) and at least half of the variation recommended by international experts (0.12%–0.16%).^[8]^ This finding was especially surprising because a major driver for the recommendation to individualize HbA1c goals is due to the increased mortality seen in patients with a high risk for or pre-existing cardiovascular disease who were randomized to intensive glycemic control in the ACCORD study.^[2]^ It is uncertain whether physicians are knowingly ignoring heart disease history in their decisions about glycemic goals or whether there are other unconscious biases influencing their choices. In contrast, hypoglycemia history has been previously reported to be a clinical factor that physicians use when deciding to discontinue or down-titrate sulfonylurea history.^[12]^

Among our physician sample, about half of respondents who individualized glycemic goals reported that this process was challenging. Physicians reported numerous challenges to individualizing HbA1c goals, especially a lack of time, confusing recommendations, and patient confusion over individualized goals. Overcoming these challenges will be necessary in order for physicians to adhere to individualized care guidelines. Solutions should include shared decision making tools for selecting individualized glycemic goals and leveraging health information technology to integrate clinical decision support tools for selecting individualized glycemic goals and individualized treatment could buoy provider efforts.^[13]^

Our study has several limitations. While our response rate is good for a national physician survey, non-response bias is possible. However, respondents and non-respondents did not differ by specialty type (*P* = .40), degree type (MD vs DO, *P* = .49), or Census region (*P* = .09). Because of the limited data available in the American Medical Association Masterfile, we were not able to assess for differences in other physician characteristics. In addition, although previous studies have supported the use of experimental clinical vignettes to assess physician behavior,^[14–17]^ the responses provided may not be consistent with actual physician behavior in clinical encounters. Moreover, this study only included physicians and did not survey other types of healthcare providers that provide routine care for patients with type 2 diabetes. The previous literature is unclear as to whether the decision-making process for glycemic goals differs significantly between physician and non-physician primary care providers.^[18]^ Also, to minimize respondent survey burden, we could not explore all of the variables important for selecting individualized goals and had to select particular ages and number of hypoglycemic events, so our findings may not generalize to other variables. There is also a concern that our survey results may be outdated and not reflect current practice. However, over the last 3 years old, the landscape of diabetes care has not changed significantly with respect to glycemic goals. The American Diabetes Association still recommends that many people should have an A1C<7% and that individualized goals should be considered for people with advanced life expectancy, high levels of comorbidity, a history of diabetes complications, and long diabetes duration. There also have not been any major studies that have contradicted these recommendations, and there has been no clinical evidence that diabetes control has changed at all in the last decade. Therefore, we believe that our survey results from 3 years ago likely reflect how physicians are practicing today. Our survey has several strengths. Our response rate was very good for a physician mail survey and the factorial vignettes allowed for the detection of the independent effects of age, heart disease risk, and hypoglycemia history on physician glycemic goal setting.

In conclusion, in this national sample of practicing primary care and endocrinology physicians, we found that physicians may select HbA1c goals around <7.0% with less regard for important patient characteristics than recommended by experts. In order to move US physician decision-making more rapidly, it may be necessary to provide more specific care guidelines or provide clinical decision support tools to reduce physician biases for the HbA1c goal of <7.0%.

## Author contributions

**Conceptualization:** Neda Laiteerapong, Robert M Sargis, Michael T Quinn, Elbert S Huang.

**Data curation:** Neda Laiteerapong, Aviva G Nathan, Elbert S Huang.

**Formal analysis:** Neda Laiteerapong, Sandra A Ham.

**Funding acquisition:** Neda Laiteerapong.

**Investigation:** Neda Laiteerapong.

**Methodology:** Neda Laiteerapong, Sandra A Ham, Aviva G Nathan, Robert M Sargis, Michael T Quinn, Elbert S Huang.

**Project administration:** Aviva G Nathan.

**Supervision:** Neda Laiteerapong.

**Writing – original draft:** Neda Laiteerapong.

**Writing – review & editing:** Neda Laiteerapong, Sandra A Ham, Aviva G Nathan, Robert M Sargis, Michael T Quinn, Elbert S Huang.

## Supplementary Material

Supplemental Digital Content

## Supplementary Material

Supplemental Digital Content
