# Supplementary material for: National physician survey on glycemic goals and medical decision making for patients with type 2 diabetes
Source: Medicine (Baltimore). 2019 Dec 20;98(51):e18491. doi: 10.1097/MD.0000000000018491 (PMC6940189; doi:10.1097/MD.0000000000018491)
Supplement: Supplemental Digital Content [file medi-98-e18491-s001.docx]

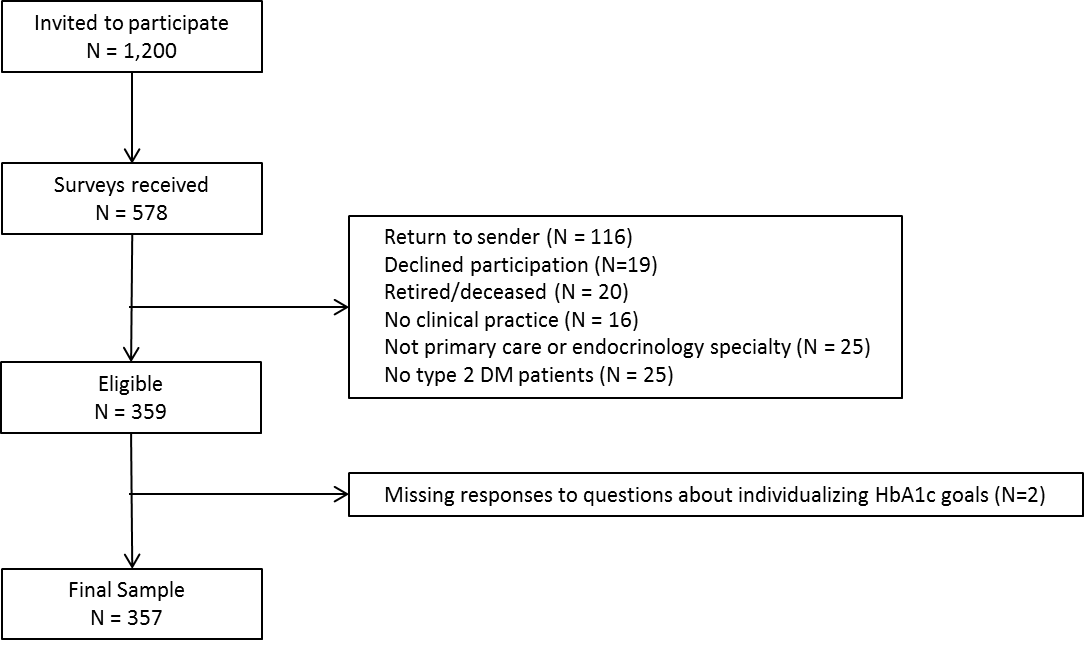


Supplemental Figure 1. Participant Flow Diagram, National Physician Survey of Type 2 Diabetes Care Practices, 2016.
