# Supplementary material for: National physician survey on glycemic goals and medical decision making for patients with type 2 diabetes
Source: Medicine (Baltimore). 2019 Dec 20;98(51):e18491. doi: 10.1097/MD.0000000000018491 (PMC6940189; doi:10.1097/MD.0000000000018491)
Supplement: Supplemental Digital Content [file medi-98-e18491-s002.docx]

Supplemental Table 1. Overall sample characteristics and by usual hemoglobin A_1c_ (HbA_1C_) goal.

|  | **Total** |  | **HbA_1C_<6.5%** | **HbA_1C_<7.0%** | **HbA_1C_<7.5 or HbA_1C_<8.0%** | **HbA_1C_ goal**  **depends on patient characteristics** |  |
| --- | --- | --- | --- | --- | --- | --- | --- |
| **Characteristic** | **N (%)** |  | **N (%)** | **N (%)** | **N (%)** | **N (%)** | **P-value** |
| Total | 357 (100.0) |  | 59 (16.5) | 197 (55.2) | 22 (6.2) | 79 (22.1) |  |
| Physician characteristic |  |  |  |  |  |  |  |
| Age, years |  |  |  |  |  |  | 0.33 |
| 30-39 | 45 (12.6) |  | 4 (6.8) | 25 (12.7) | 2 (9.1) | 14 (17.7) |  |
| 40-49 | 103 (28.9) |  | 15 (25.4) | 54 (27.4) | 5 (22.7) | 29 (36.7) |  |
| 50-64 | 149 (41.7) |  | 32 (54.2) | 79 (40.1) | 10 (45.5) | 28 (35.4) |  |
| ≥65 | 43 (12.0) |  | 6 (10.2) | 26 (13.2) | 4 (18.2) | 7 (8.9) |  |
| Gender |  |  |  |  |  |  | 0.049 |
| Female | 139 (38.9) |  | 17 (28.8) | 79 (40.1) | 5 (22.7) | 38 (48.1) |  |
| Male | 218 (61.1) |  | 42 (71.2) | 118 (59.9) | 17 (77.3) | 41 (51.9) |  |
| Years in practice |  |  |  |  |  |  | 0.03 |
| <20 | 168 (47.1) |  | 25 (42.4) | 86 (43.7) | 8 (36.4) | 49 (62.0) |  |
| ≥20 | 177 (49.6) |  | 34 (57.6) | 100 (50.8) | 14 (63.6) | 29 (36.7) |  |
| Race/ethnicity |  |  |  |  |  |  | 0.89 |
| White, non-Hispanic | 211 (59.1) |  | 33 (55.9) | 114 (57.9) | 15 (68.2) | 49 (62.0) |  |
| Asian | 74 (20.7) |  | 12 (20.3) | 43 (21.8) | 3 (13.6) | 16 (20.3) |  |
| Other | 68 (19.0) |  | 14 (23.7) | 37 (18.8) | 3 (13.6) | 14 (17.7) |  |
| Specialty |  |  |  |  |  |  | 0.002 |
| Primary care physician | 191 (53.5) |  | 40 (67.8) | 106 (53.8) | 15 (68.2) | 30 (38.0) |  |
| Endocrinologist | 164 (45.9) |  | 19 (32.2) | 89 (45.2) | 7 (31.8) | 49 (62.0) |  |
| Professional activity |  |  |  |  |  |  | 0.70 |
| No research | 334 (93.6) |  | 54 (91.5) | 187 (94.9) | 20 (90.9) | 73 (92.4) |  |
| Any research | 23 (6.4) |  | 5 (8.5) | 10 (5.1) | 2 (9.1) | 6 (7.6) |  |
| Burnout* |  |  |  |  |  |  | 0.87 |
| Yes | 110 (30.8) |  | 16 (27.1) | 62 (31.5) | 6 (27.3) | 26 (32.9) |  |
| No | 247 (69.2) |  | 43 (72.9) | 135 (68.5) | 16 (72.7) | 53 (67.1) |  |
| Setting |  |  |  |  |  | 0.02 |  |
| Other settings | 260 (72.8) | 52 (88.1) | 141 (71.6) | 13 (59.1) | 54 (68.4) |  |  |
| Hospital-based clinic | 97 (27.2) | 7 (11.9) | 56 (28.4) | 9 (40.9) | 25 (31.6) |  |  |
| Type of practice |  |  |  |  |  | 0.003 |  |
| Single specialty | 188 (52.7) | 42 (71.2) | 103 (52.3) | 12 (54.5) | 31 (39.2) |  |  |
| Multi-specialty group | 163 (45.7) | 16 (27.1) | 90 (45.7) | 10 (45.5) | 47 (59.5) |  |  |
| Urbanicity |  |  |  |  |  | 0.19 |  |
| Suburban or rural | 198 (55.5) | 39 (66.1) | 106 (53.8) | 9 (40.9) | 44 (55.7) |  |  |
| Urban | 159 (44.5) | 20 (33.9) | 91 (46.2) | 13 (59.1) | 35 (44.3) |  |  |
| Estimated number of patients |  |  |  |  |  | 0.40 |  |
| <=1000 | 138 (38.7) | 20 (33.9) | 73 (37.1) | 11 (50.0) | 34 (43.0) |  |  |
| >1000 | 212 (59.4) | 39 (66.1) | 119 (60.4) | 10 (45.5) | 44 (55.7) |  |  |
| Private insurance among payer mix |  |  |  |  |  | 0.48 |  |
| 76-100% | 14 (3.9) | 2 (3.4) | 6 (3.0) | 0 (0.0) | 6 (7.6) |  |  |
| 51-75% | 93 (26.1) | 18 (30.5) | 54 (27.4) | 6 (27.3) | 15 (19.0) |  |  |
| 26-50% | 134 (37.5) | 23 (39.0) | 76 (38.6) | 8 (36.4) | 27 (34.2) |  |  |
| 0-25% | 97 (27.2) | 15 (25.4) | 48 (24.4) | 7 (31.8) | 27 (34.2) |  |  |
| Fee-for-service among payment methods |  |  |  |  |  | 0.44 |  |
| 76-100% | 194 (54.3) | 37 (62.7) | 101 (51.3) | 10 (45.5) | 46 (58.2) |  |  |
| 51-75% | 23 (6.4) | 5 (8.5) | 13 (6.6) | 0 (0.0) | 5 (6.3) |  |  |
| 26-50% | 23 (6.4) | 4 (6.8) | 15 (7.6) | 2 (9.1) | 2 (2.5) |  |  |
| 0-25% | 51 (14.3) | 7 (11.9) | 29 (14.7) | 6 (27.3) | 9 (11.4) |  |  |
| Unknown | 66 (18.5) | 6 (10.2) | 39 (19.8) | 4 (18.2) | 17 (21.5) |  |  |
| Managed care among insurance types |  |  |  |  |  | 0.84 |  |
| 0-50% | 207 (58.0) | 33 (55.9) | 114 (57.9) | 12 (54.5) | 48 (60.8) |  |  |
| 51-100% | 120 (33.6) | 22 (37.3) | 64 (32.5) | 9 (40.9) | 25 (31.6) |  |  |
| Patients 65 years and older |  |  |  |  |  | 0.27 |  |
| 0-50% | 215 (60.2) | 42 (71.2) | 116 (58.9) | 12 (54.5) | 45 (57.0) |  |  |
| 51-100% | 134 (37.5) | 16 (27.1) | 75 (38.1) | 10 (45.5) | 33 (41.8) |  |  |
| Non-Hispanic white among patient racial/ethnic mix |  |  |  |  |  | 0.91 |  |
| 76-100% | 86 (24.1) | 15 (25.4) | 46 (23.4) | 6 (27.3) | 19 (24.1) |  |  |
| 51-75% | 108 (30.3) | 20 (33.9) | 52 (26.4) | 8 (36.4) | 28 (35.4) |  |  |
| 26-50% | 108 (30.3) | 17 (28.8) | 64 (32.5) | 5 (22.7) | 22 (27.8) |  |  |
| 0-25% | 46 (12.9) | 6 (10.2) | 28 (14.2) | 3 (13.6) | 9 (11.4) |  |  |
| Patients with Type 1 diabetes |  |  |  |  |  | 0.66 |  |
| 0-50% | 351 (98.3) | 58 (98.3) | 192 (97.5) | 22 (100.0) | 79 (100.0) |  |  |
| 51-100% | 4 (1.1) | 1 (1.7) | 3 (1.5) | 0 (0.0) | 0 (0.0) |  |  |
| Patients with Type 2 diabetes |  |  |  |  |  | 0.006 |  |
| 0-50% | 202 (56.6) | 44 (74.6) | 106 (53.8) | 15 (68.2) | 37 (46.8) |  |  |
| 51-100% | 152 (42.6) | 15 (25.4) | 88 (44.7) | 7 (31.8) | 42 (53.2) |  |  |

* Burnout was categorized as “yes” if respondents checked any of the following: “I am definitely burning out and have one or more symptoms of burnout, e.g., emotional exhaustion.”, “The symptoms of burnout that I’m experiencing won’t go away, I think about work frustrations a lot.”, or “I feel completely burned out. I am at the point where I may need to seek help.” and was categorized as “no” if respondents indicated “I enjoy my work. I have no symptoms of burnout.” or “I am under stress and don’t always have as much energy as I did, but I don’t feel burned out.”
